# FOXO1 deletion in keratinocytes improves diabetic wound healing through MMP9 regulation

**DOI:** 10.1038/s41598-017-10999-3

**Published:** 2017-09-05

**Authors:** Chenying Zhang, Jason Lim, Hyeran Helen Jeon, Fanxing Xu, Chen Tian, Fang Miao, Alhassan Hameedaldeen, Dana T. Graves

**Affiliations:** 1 Department of Preventive Dentistry, Peking University School and Hospital of Stomatology, Beijing, China; 2 Department of Periodontics, School of Dental Medicine, University of Pennsylvania, Philadelphia, PA USA; 3 Department of Orthodontics, School of Dental Medicine, University of Pennsylvania, Philadelphia, PA USA; 4 Shanxi Province People’s Hospital, Taiyuan, Shanxi province 030012 China

## Abstract

Keratinocyte migration is a key aspect of re-epithelialization during wound healing. Matrix metalloproteinase 9 (MMP9) contributes to this process and deficiencies in the MMP9 lead to impaired healing. Inappropriate expression of MMP9 also contributes to impaired re-epithelialization. Previously we demonstrated that FOXO1 was activated in wound healing but to higher levels in diabetic wounds. To address mechanisms of impaired re-epithelialization we examined MMP9 expression *in vivo* in full thickness dermal scalp wounds created in experimental K14.Cre^**+**^.*Foxo1* ^*L/L*^ mice with lineage-specific Cre recombinase deletion of floxed FOXO1 and compared the results to control littermates. MMP9 was induced during wound healing but at a significantly higher level in diabetic compared to normal wounds. FOXO1 deletion substantially blocked this increase. By chromatin immunoprecipitation FOXO1 was shown to bind to the MMP9 promoter, FOXO1 overexpression increased MMP9 transcriptional activity and increased MMP9 expression stimulated by high glucose was blocked by FOXO1 deletion or FOXO1 knockdown. We also show for the first time that high glucose impairs keratinocyte migration by inducing high levels of MMP9 expression and establish that it involves FOXO1. Thus, FOXO1 drives high levels of MMP9 expression in diabetic wound healing, which represents a novel mechanism for impaired re-epithelization in diabetic wounds.

## Introduction

Chronic diabetic wounds are a common and potentially serious complication with considerable morbidity and associated financial costs. The disturbed physiologic function of epidermal keratinocytes plays a central role in the impaired wound healing in diabetes^1^. Factors involving keratinocytes that may contribute to the dysfunctional diabetic wound healing process include impaired keratinocyte migration and proliferation, chronic inflammation, chronic infections associated with defective barrier function, impaired angiogenesis, increased oxidative stress, and abnormal expression of matrix metalloproteinases (MMPs)^2–4^. Keratinocyte migration is facilitated by extracellular matrix (ECM) degradation by MMPs, but excessive MMP activity is a feature of chronic wounds and delays wound healing^5^.

MMPs are important in wound healing by modifying the wound matrix, allowing for cell migration and tissue remodeling. MMPs and their specific inhibitors, tissue inhibitors of metalloproteinases (TIMPs), act in a coordinated fashion to regulate collagen remodeling^6^. Matrix metalloproteinases 9 (MMP9), one of the most widely investigated MMPs, is a type IV collagenase known to be expressed by keratinocytes at the leading edge of the wound and promote cell migration and re-epithelialization. In the normal tissue, MMP9 are expressed at basal levels but rapidly upregulated following wounding. As wounds heal, MMP9 diminishes^7^. If normal levels of MMP9 are suppressed, epithelialization is delayed; if persistently excessive amounts of MMP9 in chronic wounds leads to the impaired healing. Thus, a balance of this bimodal MMP9 action is critical to the epithelialization process. Although there have been numerous studies on excessive MMP9 expression in diabetic wounds^3, 8–11^, relatively little is known regarding the regulation of high expression of MMP9 during diabetic wound healing.

Forkhead box O1 (FOXO1), which belongs to a large family of forkhead transcription factors, participates in a wide range of cellular processes, including cell cycle arrest, DNA repair, apoptosis, oxidative stress resistance, and glucose metabolism^12^. Recent evidence indicates that FOXO1 plays a critical role in wound healing. FOXO1 is markedly activated in the leading edge and basal layer of keratinocytes and hair follicles by 1 day after skin injury^13^. We have shown that FOXO1 promotes wound healing through the up-regulation of TGF-β1 and prevention of oxidative stress in normal skin wounds^14^. Lineage-specific FOXO1 deletion in keratinocytes interferes with keratinocyte migration in normal skin and mucosal wounds but has the opposite effect in diabetic wounds^14–16^. Moreover, FOXO1 expression in keratinocytes promotes connective tissue healing^17^ and angiogenesis (unpublished) demonstrating that FOXO1 activates keratinocytes to affect other cell types.

In the present study, we explored the role of FOXO1 in normal and diabetic skin wound healing through induction of an important downstream target gene, MMP9, which must be carefully regulated to facilitate keratinocyte migration and re-epithelialization. We demonstrate that high expression of MMP9 in diabetic wounds *in vivo* and high glucose *in vitro* can be rescued by a lineage-specific FOXO1 deletion in keratinocytes. In addition, high glucose increases FOXO1 binding to the MMP9 promoter and high glucose stimulates MMP9 transcription in an FOXO1-dependent manner, which is blocked by the FOXO1 knockdown.

## Results

### Keratinocyte-specific FOXO1 deletion reduces MMP9 expression during wound healing

**Figure 1:**
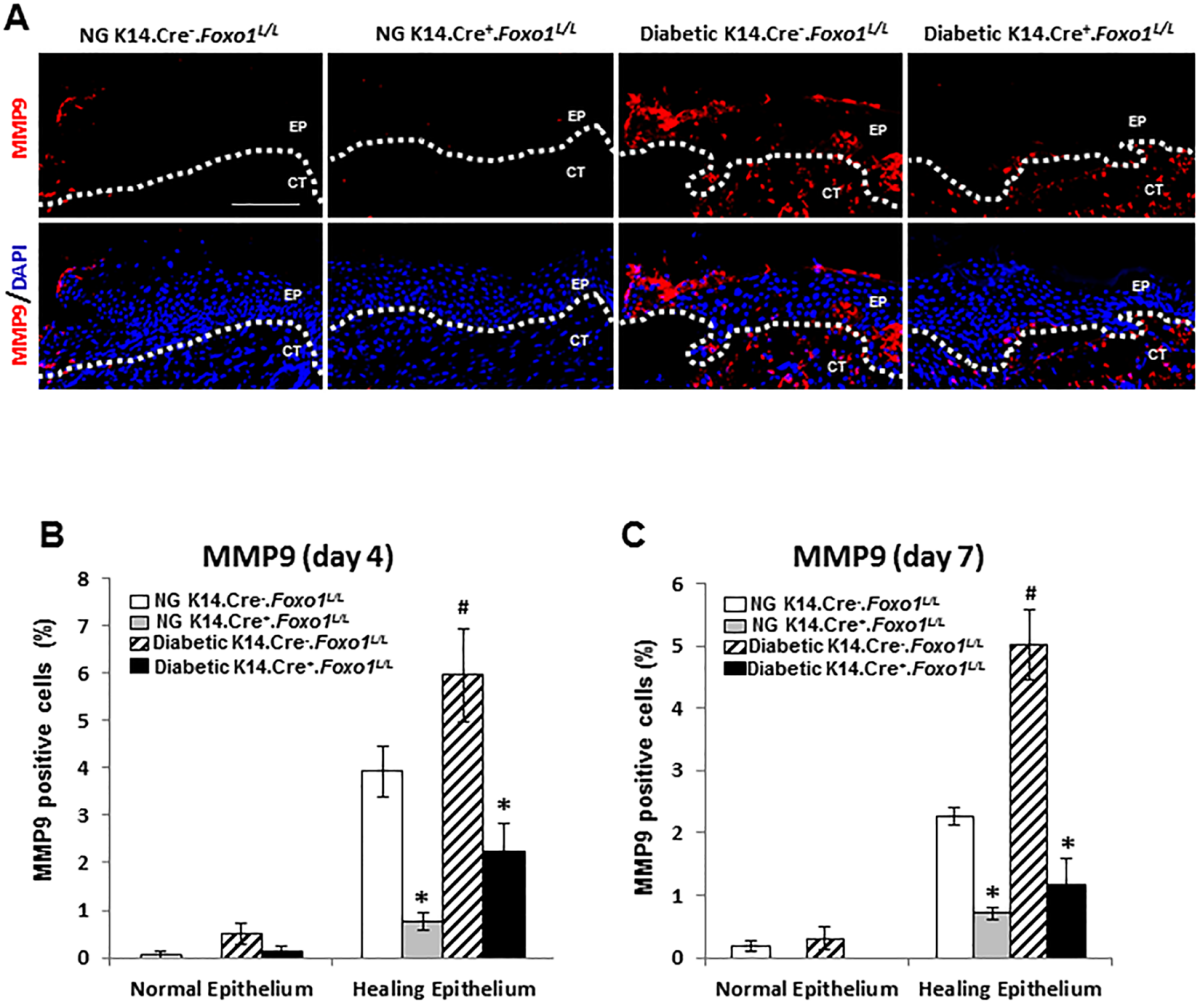
Keratinocyte-specific FOXO1 deletion reduces MMP9 expression during wound healing. Dermal wounds were created in normoglycemic (NG) and diabetic experimental (K14.Cre^+^.Foxo1^L/L^) and littermate control (K14.Cre^−^. Foxo1^L/L^) mice. (**A**) Representative images of MMP9 immunofluorescent staining with the MMP9 specific antibody on day 4 wounds. Scale bar, 100 µm. EP = epidermis, CT = connective tissues, white dashed lines demarcate the epidermis from the dermis. (**B**) MMP9 immunofluorescence analyses for day 4 wounds. (**C**) MMP9 immunofluorescence analyses for day 7 wounds. Each *in vivo* value is the mean ± SEM for n = 5–8 mice per group. *P < 0.05 vs. matched Cre^−^ group, ^**#**^ *P* < 0.05 vs. matched NG Cre- group.

Our previous study found that deletion of FOXO1 in keratinocytes of normoglycemic mice delayed wound re-epithelialization, whereas accelerated wound closure was observed after lineage-specific deletion of FOXO1 in diabetic mice^16^. A pilot study suggested that MMP9 was regulated by FOXO1 in keratinocytes (data not shown). Because MMP9 plays a critical role in both normal and diabetic wound healing^18^, we determined whether MMP9 was regulated by FOXO1 *in vivo* in transgenic mice with lineage-specific deletion of FOXO1 in keratinocytes driven by keratin-14 Cre recombinase. Small excisional skin wounds were created in experimental transgenic mice (K14.Cre^**+**^.*Foxo1* ^*L/L*^) and littermate control (K14.Cre^**−**^.*Foxo1* ^*L/L*^) mice. The impact of FOXO1 on MMP9 expression *in vivo* was examined in wounds of experimental and control mice by immunofluorescent analysis. Keratinocyte-specific FOXO1 deletion in diabetic mice resulted in an evident reduction in MMP9 level only in epithelium but not connective tissue (Fig. 1A). Upon wounding, MMP9 expression was increased over tenfold (Fig. 1B,C). MMP9 levels at the leading edge of wounds were increased by 1.5- (day 4, *P* < 0.05) and 2.2-fold (day 7, *P* < 0.05) in diabetic wounds compared with normoglycemic wounds in K14.Cre^**−**^.*Foxo1* ^*L/L*^ control mice (Fig. 1B,C). Keratinocyte-specific deletion of *Foxo1* blocked the diabetes-induced increase in MMP9 expression *in viv*o. The results indicate that the impact of diabetes on MMP9 is FOXO1 dependent.

### MMP9 expression in keratinocytes is regulated by FOXO1 *in vitro*

**Figure 2:**
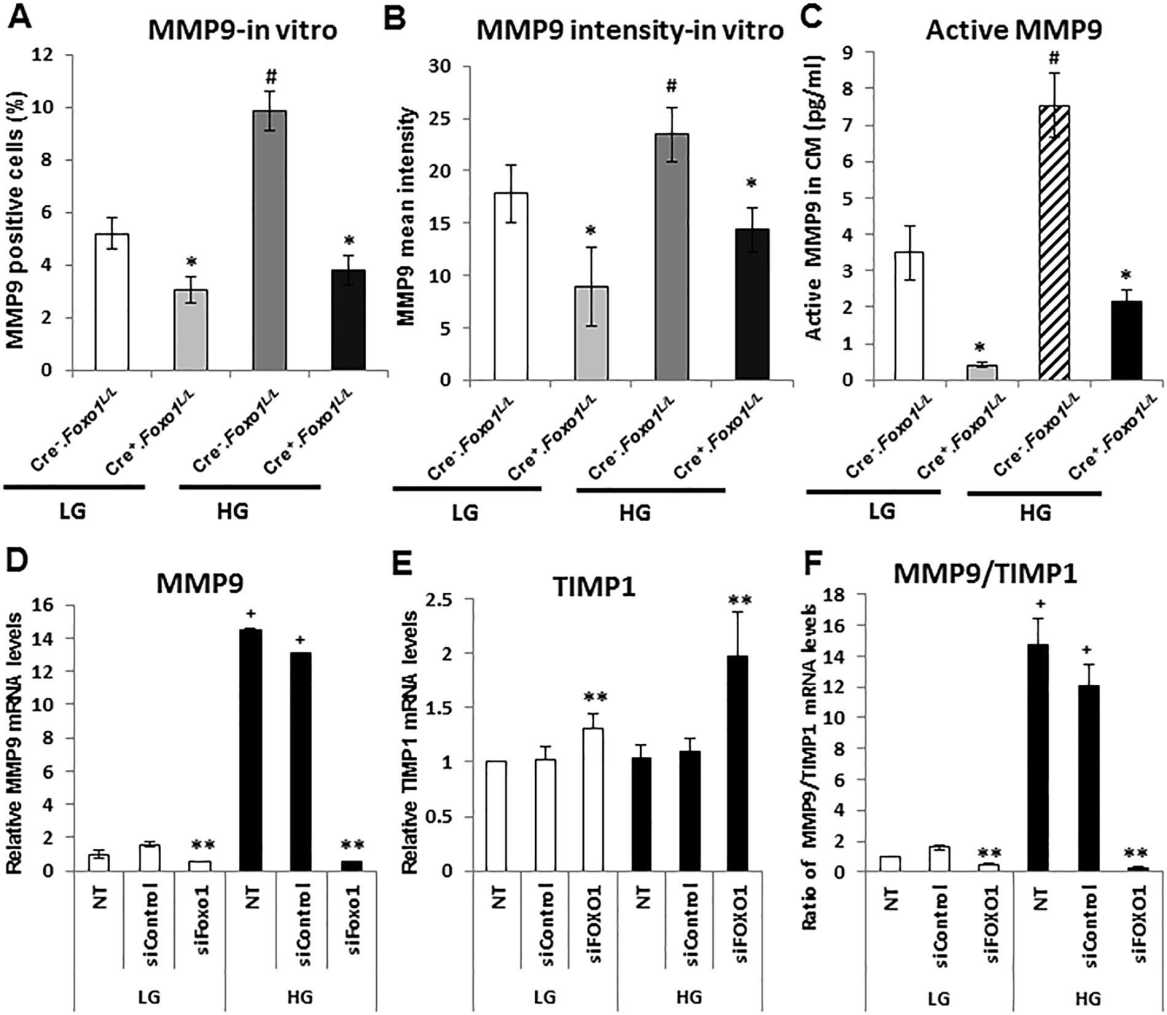
FOXO1 deletion in keratinocytes reduces MMP9 expression *in vitro*. Primary murine keratinocytes (**A-C**) from K14.Cre^**−**^.*Foxo1* ^*L/L*^ or K14.Cre^**+**^.*Foxo1* ^*L/L*^ mice and primary normal human epidermal keratinocytes (NHEK) cells (**D**–**F**) were incubated in low glucose (LG) or in high glucose (HG) media. **(A** and **B**) MMP9 immunofluorescence analyses of murine keratinocyte immune-positive cells (**A**) and MMP9 fluorescence intensity (**B**). (**C**) MMP9 ELISA analyses of conditioned media from primary murine K14.Cre^**−**^.*Foxo1* ^*L/L*^ or K14.Cre^**+**^.*Foxo1* ^*L/L*^ keratinocytes in LG or HG condition. **(D** and **E**) qRT-PCR analysis of MMP9 (**D**) and TIMP1 (**E**) mRNA levels. (**F**) The ratio of MMP9 to TIMP1 mRNA levels. Each data represents the mean ± SEM of 3 independent experiments. *P < 0.05 vs. matched Cre-, ^**#**^ *P* < 0.05 vs. LG Cre^−^ group, **P < 0.05 vs. matched scrambled siRNA, ^**+**^ *P* < 0.05 vs. matched LG group.

The effect of FOXO1 on MMP9 expression *in vitro* was further examined in primary murine keratinocytes isolated from normal and experimental mice. At the protein level, high glucose increased MMP9 expression 1.3 to 2-fold (*P* < 0.05), and this increase was blocked by *Foxo1* ablation in keratinocytes (*P* < 0.05) (Fig. 2A,B). Active MMP9 levels in conditioned medium from keratinocytes isolated from K14.Cre^**-**^.*Foxo1* ^*L/L*^ mice and K14.Cre^**+**^.*Foxo1* ^*L/L*^ mice were then determined by ELISA. The level of activated MMP9 was increased more than 2-fold by high glucose (*P* < 0.05). FOXO1 deletion in primary murine keratinocytes reduced activated MMP9 by 88% in normal glucose media and by 72% in high glucose media (*P* < 0.05) (Fig. 2C). Primary cultures of human keratinocytes were also tested *in vitro*. MMP9 mRNA levels increased 15-fold in high glucose media (*P* < 0.05) and knockdown of FOXO1 blocked this increase (*P* < 0.05) (Fig. 2D). Under normoglycemic conditions, knockdown of FOXO1 led to a slight increase in TIMP1 mRNA, an MMP inhibitor, and a 2-fold increase in TIMP1 in high glucose media (*P* < 0.05) (Fig. 2E). The ratio of MMP9/TIMP mRNA increased more than 10-fold in high glucose (*P* < 0.05) and this increase was blocked by FOXO1 knockdown (*P* < 0.05) (Fig. 2F). Thus, FOXO1 played a prominent role in inducing MMP9 and inhibiting TIMP1, particularly when keratinocytes were incubated in high glucose.

**Figure 3:**
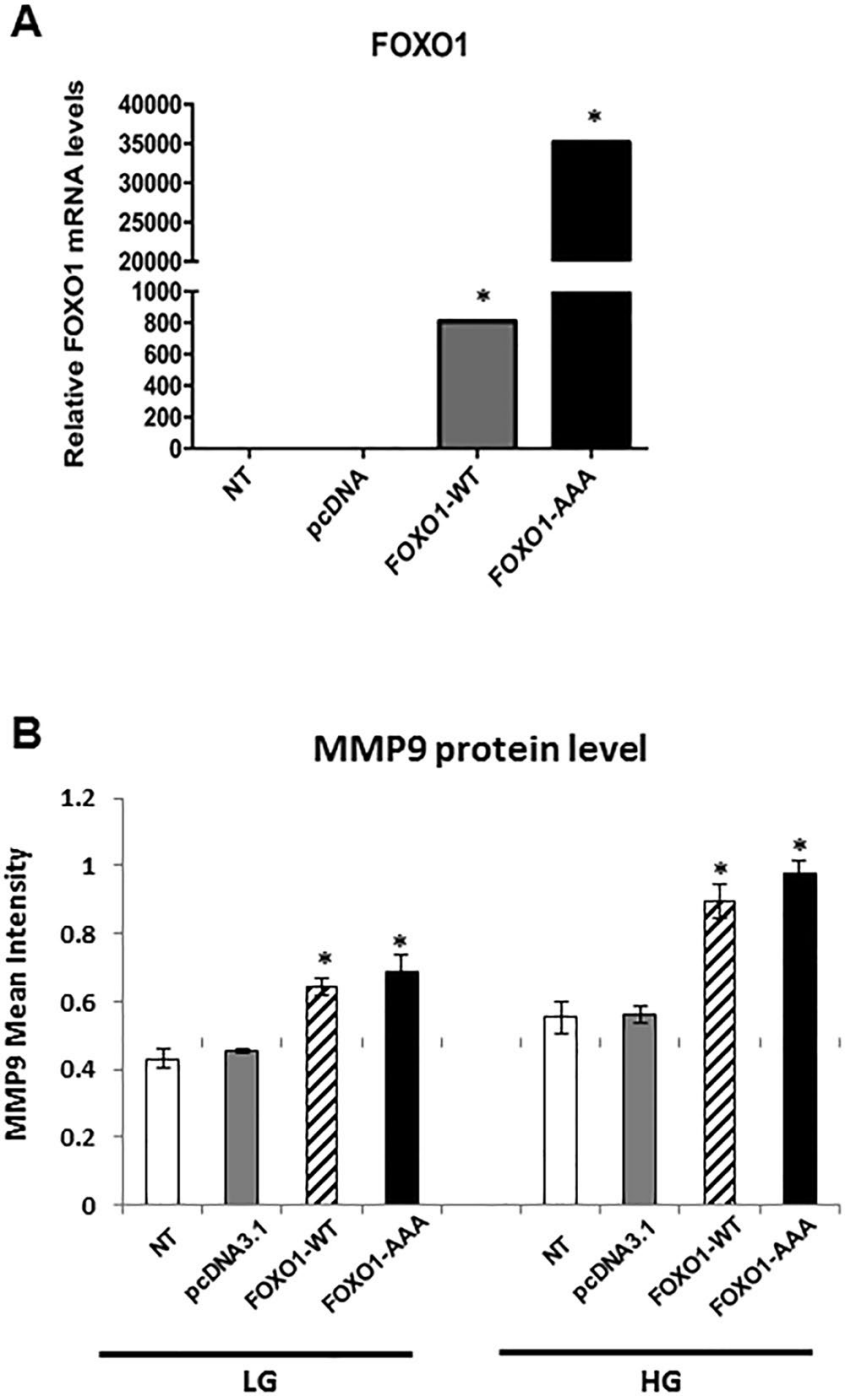
FOXO1 regulates the protein level of MMP9 in keratinocytes. (**A**) FOXO1 mRNA analyses in NHEK cells after transfection with control plasmid, wild-type FOXO1 plasmid (FOXO1-WT), or constitutively active FOXO1 plasmid (FOXO1-AAA) in LG media. (**B**) MMP9 fluorescence intensity in NHEK cells after transfection with control plasmid, wild-type FOXO1 plasmid (FOXO1-WT), or constitutively active FOXO1 plasmid (FOXO1-AAA) in LG or HG media. *P < 0.05 vs. matched control plasmid.

The effect of FOXO1 over-expression on MMP9 levels was examined. FOXO1 mRNA increased more than 800-fold (*P* < 0.05) after overexpression of wild-type FOXO1 (FOXO1-WT), while over-expression of constitutively active FOXO1 (FOXO1-AAA) resulted in a much greater increase in FOXO1 mRNA (*P* < 0.05) (Fig. 3A). Over-expression of FOXO1 triggered a 1.6-fold increase in MMP9 protein levels in human keratinocytes in normal or high glucose conditions (*P* < 0.05) (Fig. 3B). The above results indicate that MMP9 levels are regulated by FOXO1 both in normal glucose condition and high glucose.

### Inhibition of MMP9 rescues keratinocyte migration in a FOXO1-dependent manner in high glucose but not low glucose conditions

**Figure 4:**
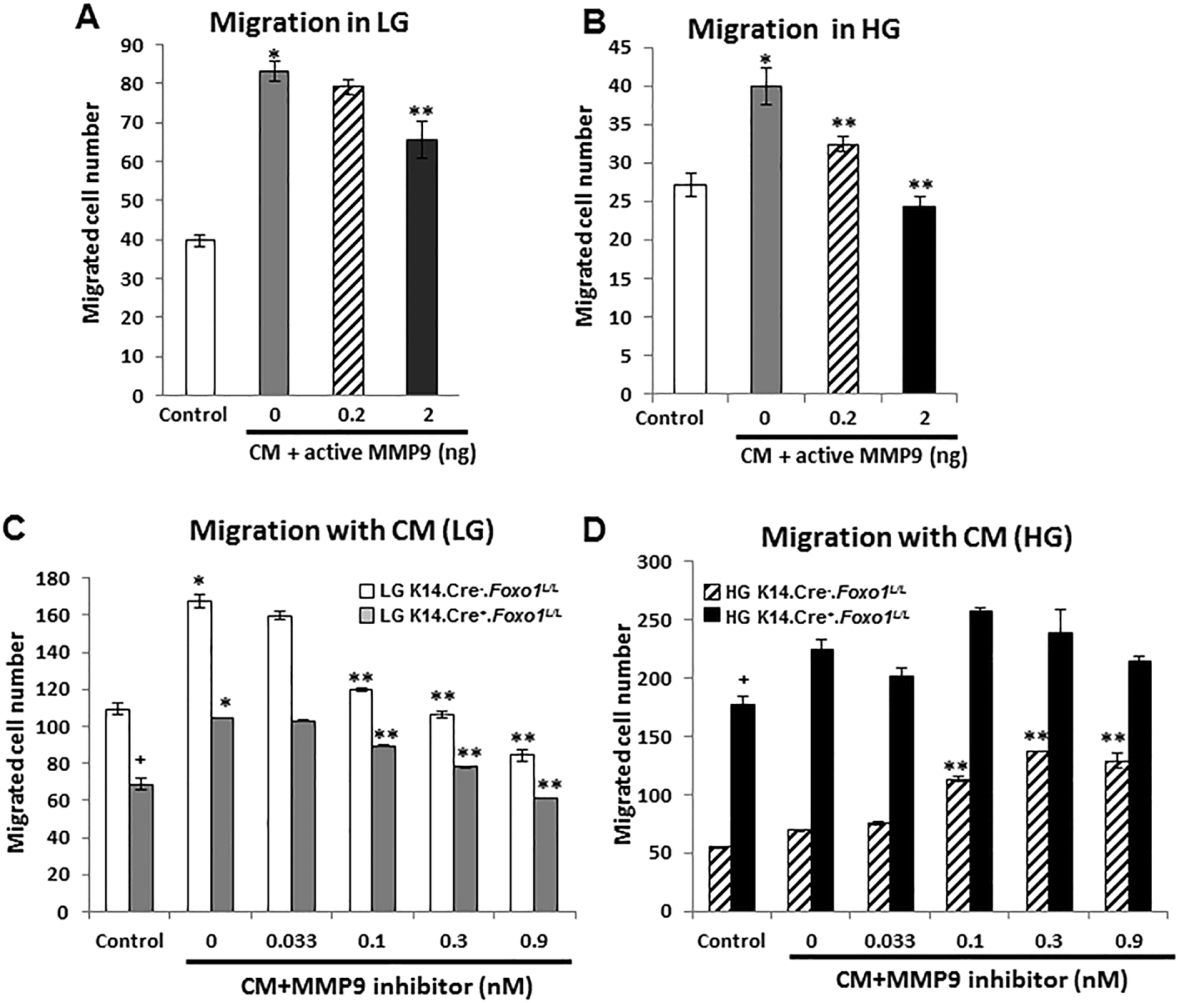
Inhibition of MMP9 rescues keratinocyte migration in a FOXO1 dependent manner in high glucose but not low glucose conditions. (**A** and **B**) Migration was measured by transwell assay for NHEK cells in the presence of conditioned media (CM) with or without indicated dose of active MMP9 protein in LG (**A**) or HG (**B**) conditions. (**C**) Migration was measured by transwell assay for primary murine keratinocytes isolated from K14.Cre^−^.*Foxo1* ^*L/L*^ or K14.Cre^+^.*Foxo1* ^*L/L*^ mice in LG condition with CM. (**D**) Migration was measured by transwell assay for primary murine keratinocytes isolated from K14.Cre^−^.*Foxo1* ^*L/L*^ or K14.Cre^+^.*Foxo1* ^*L/L*^ mice in HG condition with CM. Data represent the mean ± SEM of 3 independent experiments. *P < 0.05 vs. matched control group, **P < 0.05 vs. matched CM control group, ^+^ *P* < 0.05 vs. matched Cre- group.

Since MMP9 is known to play an instrumental role in keratinocyte migration we examined this further^18^. Migration of human keratinocytes was reduced in high glucose medium by 30% compared to cells in normal glucose medium (Fig. 4A,B). Treatment with conditioned medium from primary human keratinocytes in low or high glucose condition induced a 1.5 to 2-fold’s increase (*P* < 0.05) (Fig. 4A,B). The addition of activated MMP9 protein reduced keratinocyte migration in a concentration-dependent manner in both low and high glucose (*P* < 0.05) (Fig. 4A,B). The effect of MMP9 on the migration of primary murine keratinocytes from experimental (K14.Cre^**+**^.*Foxo1* ^*L/L*^) and control (K14.Cre^**−**^.*Foxo1* ^*L/L*^) mice was then examined. Keratinocyte migration was reduced about 40% by FOXO1 deletion in normal glucose condition (Fig. 4C). MMP9 inhibitor dose-dependently decreased keratinocyte migration in normal glucose condition (*P* < 0.05) whether FOXO1 was normal or not, demonstrating that in low glucose conditions the amount of MMP9 was optimal since the reduction of MMP9 reduced migration (Fig. 4C). The opposite result was obtained in conditioned medium from keratinocytes incubated in high glucose. Conditioned medium from FOXO1 deleted keratinocytes in high glucose stimulated migration 3-fold more than conditioned medium from control keratinocytes (*P* < 0.05) (Fig. 4D). Moreover, MMP9 inhibitor significantly enhanced the stimulatory effect of conditioned medium from control keratinocytes incubated in high glucose (*P* < 0.05) (Fig. 4D). The rescue effect of MMP9 inhibitor disappeared in conditioned medium from FOXO1 deleted keratinocytes in high glucose. The result indicates that the negative effect of high glucose on keratinocyte migration is due in part to high levels of MMP9 mediated by FOXO1 in high glucose.

### FOXO1 is recruited to the MMP9 promoter and transactivates its expression

**Figure 5:**
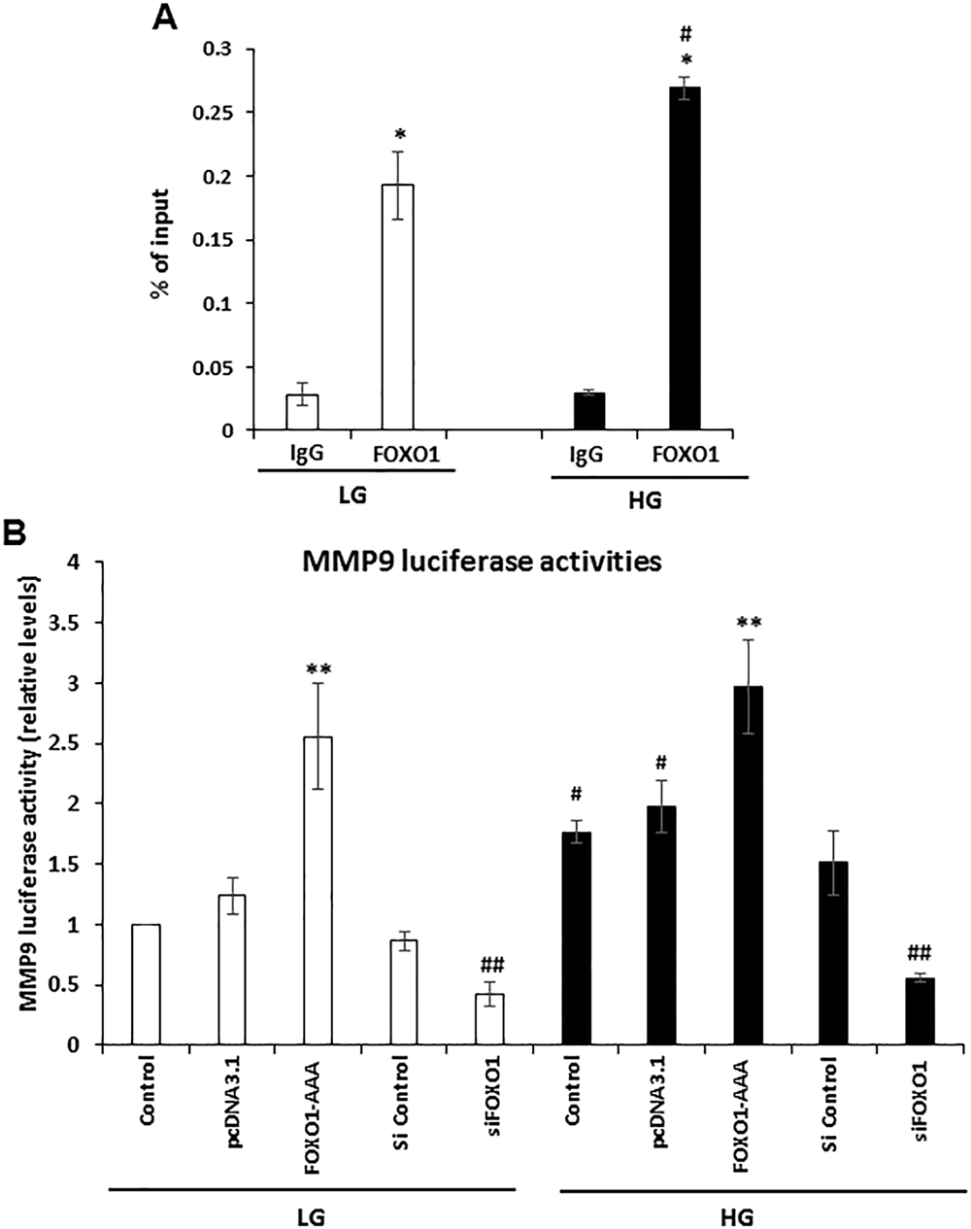
FOXO1 is recruited to the MMP9 promoter and transactivates its expression. (**A**) ChIP assays for the binding of FOXO1 to the MMP9 promoter in NHEK cells in LG and HG conditions. ChIP-enriched DNA was quantified by qRT-PCR and values expressed as a percentage of input DNA. (**B**) NHEK cells were co-transfected with a control pcDNA3.1 vector or a vector that expresses constitutively active FOXO1 (FOXO1-AAA), control siRNA, or siRNA specific to FOXO1, together with a MMP9 reporter plasmid. *Renilla* luciferase reporter was used as an internal control. Luciferase activity was measured 36 h after transfection. Data represent the mean ± SEM of 3 independent experiments. *P < 0.05 vs. matched IgG control, ^**#**^ *P* < 0.05 vs. matched LG group, **P < 0.05 vs. matched control plasmid, ^**##**^ *P* < 0.05 vs. matched scrambled siRNA.

Whether FOXO1 regulates MMP9 transcription was further explored. Chromatin immunoprecipitation (ChIP) assay demonstrated that FOXO1 binds to the MMP9 promoter and that high glucose induces a 40% higher level of FOXO1 binding to the MMP9 promoter compared to low glucose (*P* < 0.05) (Fig. 5A). To determine whether FOXO1 can transactivate the MMP9 to increase promoter activity, we examined an MMP9 luciferase reporter in primary human keratinocytes. High glucose induced a significant 77% increase in MMP9 promoter activity in keratinocytes compared with low glucose (*P* < 0.05) (Fig. 5B). Over-expression of constitutively active FOXO1 (FOXO1-AAA) resulted in a 1.5 to 2-fold increase in MMP9 promoter activity, whereas FOXO1 silencing produced a 50% to 63% reduction in MMP9 promoter activity (*P* < 0.05) (Fig. 5B). Taken together, these results indicate that FOXO1 directly binds and transactivates MMP9 promoter activity and conforms that MMP9 is a downstream target of FOXO1. Both the ChIP assays and the reporter assays included the predicted single FOXO1 consensus element located at −784 to −774bp in the human MMP9 promoter. In addition, high glucose directly increased MMP9 promoter activity which wass blocked by the FOXO1 knockdown. Collectively the results indicate that high glucose increases FOXO1 binding to the MMP9 promoter and high glucose stimulates MMP9 transcription in a FOXO1-dependent manner.

## Discussion

We previously reported that FOXO1 has a positive impact on healing in normal mice whereas in diabetic mice FOXO1 has the opposite effect and impedes re-epithelialization^14–16^. Results presented here investigated a mechanism through which FOXO1 facilitates keratinocyte migration in normal wound healing and impairs it in diabetic conditions. In our study, we found that FOXO1 regulates MMP9 expression during normal and diabetic dermal scalp healing. Diabetes *in vivo* or high glucose *in vitro* increased MMP9 expression by keratinocytes. Deletion of FOXO1 in keratinocytes blocked MMP9 expression. Keratinocytes in high glucose had impaired migration that was rescued with an MMP9 inhibitor and also rescued by FOXO1 deletion or RNAi knockdown. The results indicate that FOXO1 is needed for MMP9 production by keratinocytes in normal healing but diabetic wound healing leads to high levels of MMP9. High levels of MMP9 in turn negatively affect keratinocyte migration and may retard re-epithelialization.

MMP9 activity is necessary for normal wound healing. Blocking MMP9 activity in normal wounds delays re-epithelialization *in vivo* and interferes with keratinocyte mobility *in vitro* ^18, 19^. Thus, keratinocyte migration and re-epithelialization are dependent on MMP9^20^. We found that FOXO1 regulates MMP9 expression in keratinocytes in normal wound healing as shown by a significant reduction in K14.Cre^**+**^.*Foxo1* ^*L/L*^ mice compared to littermate control mice. Primary keratinocytes isolated from experimental K14.Cre^**+**^.*Foxo1* ^*L/L*^ mice cultured in media with normal glucose had significantly less MMP9 expression compared to matched control keratinocytes. In contrast, FOXO1 overexpression increased MMP9 mRNA and protein levels. In addition, we found that FOXO1 directly binds and transactivates MMP9 promoter activity, indicating that MMP9 is a downstream target of FOXO1. These results are the first demonstration that FOXO1 can directly promote MMP9 transcription and regulate MMP9 expression *in vivo* during wound healing. They are consistent with a previous report that FOXO1 mediates IL-1 induced MMP9 expression in myometrial cells^21^. Moreover, the finding that FOXO1 regulates expression of MMP9 adds to our previous report that FOXO1 directs the behavior of keratinocytes in normal wounds to promote the healing process^14^.

We found that an MMP9 inhibitor significantly reduced the ability of conditioned media from wild-type keratinocytes to stimulate keratinocyte migration. In contrast, an MMP9 inhibitor had minimal effect on migration of FOXO1 deleted keratinocytes *in vitro*. This suggests that when FOXO1 is deleted, the MMP9 expression is low so that an MMP9 inhibitor has little effect. The regulation of MMP9 by FOXO1 may also be important in events where keratinocyte-derived MMP9 is thought to play a role such as granulation tissue remodeling^22^. The latter agrees well with our recent report that FOXO1 in keratinocytes promotes connective tissue healing^17^.

Although it is recognized that MMP9 is expressed at high levels in keratinocytes in diabetic wounds^23^ and that high glucose levels interfere with keratinocyte migration^24^ the link between hyperglycemia, MMP9 expression, and impaired keratinocyte migration has not been previously reported. An MMP9 inhibitor incubated with keratinocytes in high glucose significantly enhanced the stimulatory effect of conditioned medium from control keratinocytes, whereas the rescue effect of MMP9 inhibitor disappeared in conditioned medium from FOXO1 deleted keratinocytes in high glucose. The result indicates that the negative effect of high glucose on keratinocyte migration is partly attributable to high levels of MMP9 expressed by keratinocytes which is mediated by FOXO1. Moreover, the addition of activated MMP9 significantly reduced keratinocyte migration in high glucose media. This is the first demonstration that high glucose can impair keratinocyte migration through the expression of high levels of MMP9.

Hyperglycemia in diabetic animals *in vivo* enhanced MMP9 expression compared with normoglycemic wounds. *In vitro*, high glucose increased the mRNA levels of MMP9 as well as activated MMP9, both of which were blocked by FOXO1 ablation in keratinocytes. Thus, a high glucose environment leads to high levels of MMP9 expression in keratinocytes, which is blocked by FOXO1 deletion or by RNAi. Accumulating evidence suggests that high levels of activated FOXO1 are linked to several diabetic complications^15, 16, 25–28^. We have previously reported that high glucose significantly enhances the binding of FOXO1 to the CCL20 promoter but for other genes such as TGFβ1, high glucose interferes with FOXO1 binding to the promoter^15, 16^. However, in studies reported here, we did not find clear-cut evidence that high glucose significantly affected binding of FOXO1 to the MMP9 promoter although FOXO1 deletion clearly blocked the effect of high glucose in enhancing MMP9 expression. Thus, we can speculate that other transcription factors may participate in high glucose-induced MMP9 expression in addition to FOXO1.

In summary, our findings define for the first time a novel function for FOXO1 in the diabetic wound healing process through MMP9 regulation. Our results have demonstrated that the activation of MMP9 is downstream of FOXO1. Understanding the mechanism responsible for the impaired keratinocytes migration in diabetes by characterizing the excessive MMP9 expression mediated by FOXO1 will help set the basis for clinical application of a FOXO1 antagonist in treating diabetic wounds.

## Materials and Methods

### Animals and induction of diabetes

Experiments were approved by the University of Pennsylvania Institutional Animal Care and Use Committee and all experiments were performed in accordance with relevant guidelines and regulations. Mice with floxed Foxo1 were provided by R.A. DePinho (MD Anderson Cancer Center, Houston, TX) as previously described^29^. Mice expressing Cre recombinase under the control of keratin 14 promoter (K14-Cre; strain Tg(KRT14-cre)1Amc/J) were obtained from the Jackson Laboratory (Bar Harbor, ME) and bred with floxed FOXO1 mice to produce experimental (K14.Cre^+^ *.Foxo1* ^*L/L*^) and littermate control (K14.Cre^−^.*Foxo1* ^*L/L*^) mice. Experiments were performed with adult mice 16–20 wk old. Type 1 diabetes was induced by i.p. injection of multiple low dose streptozotocin (40 mg/kg; Sigma-Aldrich) in 10 mM citrate buffer daily for 5 days and control mice were injected with citrate buffer alone. Peripheral blood was assessed with an electronic glucometer and experiments were initiated when glucose levels exceeded 220 mg/dl for at least 3 weeks.

### Skin wounding experiment

Mice were anaesthetized with ketamine (80 mg/kg) and xylazine (5 mg/kg) administered i.p. The skin overlying the calvarial bone was shaved and cleansed with isopropyl alcohol. Two full thickness excisional wounds were created with a 2mm sterile biopsy punch as described previously^30^. In this model, scalp wounds heal by the production of new connective tissue more than by contraction, making the repair process more similar to human wounds compared to dorsal skin wounds in mice that heal primarily by contraction^14, 30, 31^. Moreover, the effect of FOXO1 deletion in keratinocytes is similar in scalp and dorsal skin wounds (unpublished data). Mice were euthanized day 4 and 7 after wounding.

### Immunohistochemistry in histological sections

The wounded dermal specimen and attached calvarial bone were fixed in 4% paraformaldehyde for 24 h, decalcified in 10% EDTA solution, and embedded in paraffin. 4-μm paraffin sections were prepared and examined by immunofluorescence. Sections were subjected to antigen retrieval, 10 mM of citric acid, pH 6.0, at 120 °C, blocked with nonspecific binding blocking buffer (Millipore), and incubated with primary antibody followed by the biotinylated secondary antibody (Vector Laboratories). Sections were subsequently incubated with avidin-biotin peroxidase enzyme complex (Vector Laboratories) and followed by tyramide signal amplification (PerkinElmer). Nuclei were stained with DAPI. Primary antibodies used were FOXO1 (rabbit; Santa Cruz Biotechnology, Inc.) and MMP9 (rabbit; Abcam). Images were captured at 100x, 200x, and 400x magnification with a fluorescence microscope (ECLIPSE 90i; Nikon) with the same exposure time for experimental and negative control groups and analyzed with NIS Elements AR image analysis software (Nikon). The number of immune-positive cells divided by the number of DAPI-positive cells was used to obtain the percent immune-positive cells.

### Cell culture

Primary normal human epidermal keratinocytes (NHEK) were purchased from Lonza and cultured in KGM-2 growth medium with growth supplements (Lonza) and antibiotics (Life Technologies). Primary mouse epidermal keratinocytes were isolated from the neonates (0–2 days old) of experimental (K14.Cre^+^. *Foxo1* ^*L/L*^) and control (K14.Cre^−^. *Foxo1* ^*L/L*^) mice. Briefly, full thickness mouse skin was obtained and incubated with 2.5 U/mL Dispase II (Roche Diagnostics) overnight at 4 °C. The dermis was separated from the epidermis by 0.1% trypsin and 0.02% EDTA in PBS for 15 minutes at 37 °C. Keratinocytes from the epidermis were cultured in KGM-2 growth medium containing antibiotics. Cell cultures were maintained in a 5% CO_2_ humidified incubator at 37 °C. Keratinocytes were passaged in KGM-2 growth media with supplements including standard insulin (8.6 × 10^−7^M). For assays cells were transferred to KGM-2 media with supplements except for the amount of insulin was reduced 100 fold unless otherwise stated, i.e. low insulin represents 1% of the amount of insulin in standard keratinocyte growth media. In some experiments, no insulin was present as indicated below.

### Transfection and RNA analysis

ON-TARGETplus SMARTpool siRNAs specific for human FOXO1 and control siRNA (ON-TARGETplus Non-targeting Control Pool) were obtained from Dharmacon and transfection was performed using GenMute siRNA Transfection Reagent (SignaGen Labs). Transient transfection with plasmid DNA was carried out using Lipofectin (Invitrogen) according to the manufacturer’s instructions. For mRNA analysis NHEK cells transfected with FOXO1 siRNA or FOXO1 plasmid were isolated using an RNeasy kit (Qiagen). Reverse transcription was performed using the High Capacity cDNA Reverse Transcription kit (Applied Biosystems). Real-time quantitative PCR (qPCR) was performed with the Taqman system (Roche Diagnostics). Results were normalized with values obtained from ribosomal protein RPL32, a ribosomal protein. Experiments were repeated three to four times.

### Immunofluorescence analysis *in vitro*

Primary keratinocytes isolated from K14.Cre^**−**^. *Foxo1* ^*L/L*^ and K14.Cre^**+**^. *Foxo1* ^*L/L*^ mice or NHEK cells transfected with wild-type or constitutively active FOXO1 plasmid were grown on 8-well chamber slides (Thermo Scientific) and incubated in low glucose (5 mM D-glucose) or high glucose (25 mM D-glucose) for 5 days. For immunofluorescence keratinocytes were fixed in cold methanol, permeabilized in 0.5% Triton X-100, blocked in 2% BSA, and stained with primary antibody against MMP9 (Abcam). Signal was localized with a biotinylated secondary antibody and streptavidin-conjugated Alexa-546. Cells were stained with DAPI and observed under a fluorescence microscope (Nikon). Images were captured at a magnification of 200 with the same exposure time for experimental and negative control groups.

Image analysis was performed using NIS Elements AR image analysis software (Nikon). The percentage of immune-positive cells and mean fluorescence intensity was measured. Mean fluorescence intensity was measured with a maximum fluorescence intensity set at 3,000 arbitrary units to obtain results in the linear response range. Fluorescence intensity measurements were calculated by subtracting mean fluorescence intensity values of control IgG from values obtained with each antibody.

### ELISA

Active MMP9 levels in conditioned media from keratinocytes isolated from K14.Cre^**−**^.*Foxo1* ^*L/L*^ mice and K14.Cre^**+**^.*Foxo1* ^*L/L*^ mice in low and high glucose conditions were determined by measuring the levels of active MMP9 in media using mouse MMP9 activity assay kit (Cedarlane) following the manufacture’s instruction.

### Keratinocytes transwell migration assay

Migration was assessed in a transwell assay with a polycarbonate membrane filter (Corning Costar, 6.5 mm diameter, 8 μm pore size). Briefly, 1 × 10^5^ cells were placed in the upper chamber of a transwell. After 6 hours cells remaining on the upper surface of the membrane were removed with cotton swabs and migrated cells on the lower surface of the membrane were stained with DAPI and counted by fluorescence microscopy. Assays were performed in triplicates.

In some cases, NHEK cells were grown in low glucose (5 mM) or high glucose (25 mM) medium for 5 days and conditioned medium (CM) from the last 48 hours was collected. 1 × 10^5^ cells were added to the upper chamber of a transwell chamber (Corning) in fresh medium or conditioned medium with or without activated MMP9 protein to measure the effect on keratinocyte migration. In some cases, conditioned medium in high glucose conditions was first collected from primary keratinocytes isolated from K14.Cre^**−**^.*Foxo1* ^*L/L*^ mice and K14.Cre^**+**^.*Foxo1* ^*L/L*^ mice. Then the cells were pre-incubated with indicated dose of MMP9 inhibitor (Millipore), and incubated in normal medium or conditioned medium collected above plus MMP9 inhibitor, which was added to the upper chamber of the transwell.

### ChIP and Luciferase Reporter Assays

NHEK cells were incubated in high glucose for 5 d. ChIP assays were performed using ChIP-IT kit (Active Motif, Carlsbad, CA) following the manufacturer’s instructions. To precipitate FOXO1, anti-FOXO1 antibody (Santa Cruz Biotechnology, Inc.) was used, and the quantitative real-time PCR of MMP9 promoters was performed. ChIP experiments were repeated three times with reproducible results. A single putative FOXO1 binding site was predicted by PROMO bio prediction software located at −784 to −774bp in the human MMP9 promoter (http://alggen.lsi.upc.es/cgi-bin/promo_v3/promo/promoinit.cgi?dirDB=TF_8.3). Luciferase activity was measured with a Dual Luciferase Reporter Assay kit (Promega) according to the manufacturer’s instructions. In brief, NHEK cells were co-transfected with MMP9 Luciferase reporter^32^ (provided by Young Han Lee, Institute of Biomedical Science and Technology, Konkuk University, Korea) together with pRL-TK luciferase control vector, FOXO1-AAA plasmid that is constitutively transported to the nucleus, or pcDNA3.1 control plasmid, scrambled siRNA, or FOXO1 siRNA. 48 hours after transfection, cells were lysed, and *Firefly* and *Renilla* luciferase activities were measured using Dual Luciferase Reporter Assay kit (Promega) according to the manufacturer’s instructions. *Firefly* luciferase activities were divided by *Renilla* activities to normalize for transfection efficiency. Experiments were performed three times with similar results.

### Statistics

Statistical analysis between two groups was performed using two-tailed Student’s t-test. In experiments with multiple time points or treatments, differences between the wild-type and experimental groups were determined by one-way ANOVA with Tukey’s posthoc test. Results are expressed as the mean ± SEM. P < 0.05 was considered statistically significant.
